# Calculating Home Advantage in the First Decade of the 21th Century UEFA Soccer Leagues

**DOI:** 10.2478/hukin-2013-0054

**Published:** 2013-10-08

**Authors:** Miguel Saavedra García, Óscar Gutiérrez Aguilar, Paulo Sa Marques, Gabriel Torres Tobío, Juan J. Fernández Romero

**Affiliations:** 1 Sports Science and Physical Activity Faculty, University of A Coruña (Spain).; 2 University Miguel Hernández of Elche (Spain).; 3 Maia Higer Institute (Portugal).

**Keywords:** Home advantage, football, UEFA, performance analysis

## Abstract

Home advantage has been studied in different sports, establishing its existence and its possible causes. This article analyzes the home advantage in soccer leagues of UEFA countries in the first part of the 21st century. The sample of 52 countries monitored during a period of 10 years allows us to study 520 leagues and 111,030 matches of the highest level in each country associated with UEFA. Home advantage exists and is significant in 32 of the 52 UEFA countries, where it equals 55.6%. A decrease can be observed in the tendency towards home advantage between the years 2000 and 2010. Values between 55 and 56 were observed for home advantage in the top ten leagues in Europe. It has also been observed that home advantage depends on the level of the league evaluated using UEFA’s 2010/11 Country coefficients. The home advantage is calculated taking into account the teams’ position and the points obtained in each of the leagues. A direct relationship was observed with the number of points gained and an inverse relationship was observed with the team position.

## Introduction

The analysis of playing at home started with the studies of Schwartz and Barsky (1977) in American sports such as basketball, ice hockey, American football and baseball. These studies, followed by Nevill and Holder (1999), determined that the advantage is real. This kind of study has been used in three types of sport. On the one hand, we have sports that use a points system with a high level of subjective discrimination on the part of the judges, such as gymnastics. Another group of sports are the ones based on objective values, like time or distance, sports such as athletics. The third group is formed by sports where judges have a certain subjective intervention, such as team sports, where referees apply the rules with different possibilities of interpretation.

The approach in this type of study has also been varied. Some have analyzed the case of playing at home in competitions with only one headquarters, with just one home side, such as the Olympic Games, taking into account both Summer and Winter Games. Balmer et al. (2001; 2003) analyzed these competitions and determined that in sports that belong to the first group, those judged mainly in a subjective manner, there was a clear existence of an advantage for home teams, thus, they achieved a very important point deviation in their favour.

The home advantage analysis in team sports has been calculated in many different sports, for example baseball (Adams and Kupper, 1994; Bray et al., 2005; Dosseville, 2007; Levernier and Barrilla, 2007), football (Carmichael and Thomas, 2005; Dosseville, 2007; Nevill et al., 1996; Pollard, 1986; 2000; 2006b; Sánchez et al., 2009; Seckin and Pollard, 2007; Thomas et al., 2004; Thomas et al., 2006), ice hockey (Agnew and Carron, 1994), basketball (Greer, 1983; Jones, 2007; Moore and Brylinsky, 1993; Varca, 1980), volleyball (Marcelino et al., 2009), rugby (Thomas et al., 2008) and handball (Gutierrez et al., 2012).

Various studies have tried to identify the factors that influence home advantage. Courneya and Carron (1992) found significant such factors as rules of the competition, public, trips made, referees and familiarity with the stadium or grounds.

Other home advantage factors analyzed were the influence of the public with regard to the number of fans, density of the crowd, proximity of them to the playing area or intensity of their support (Pollard, 2006a; Pollard and Pollard, 2005; Wolfson et al., 2005). Further factors were possible alterations caused by the journeys made by the players (Brown et al., 2002; Clarke and Norman, 1995; Pollard, 2006a), better knowledge of the playing ground, such as specific conditions or non-standard dimensions (Barnett and Hilditch, 1993; Clarke and Norman, 1995; Pollard, 1986), unfamiliar climatic conditions (Pollard et al., 2008; Seckin and Pollard, 2007) or the behaviour of the referees, in the frequency of their use of disciplinary sanctions (Glamser, 1990; Nevill et al., 1996).

The study of home advantage evaluated the existence of alterations at the physiological level (Neave and Wolfson, 2003), verifying a rise in hormonal activity in home teams. The use of different game tactics in home and away teams has also been studied, in order to determine mainly if there are any variations in the performance of the teams (Carmichael and Thomas, 2005; Seckin and Pollard, 2007; Tucker et al., 2005).

The objectives of this study were as follows: to determine and verify the home advantage in soccer in the UEFA zone in the first decade of the 21st century and its evolution during this time. We evaluated the behaviour of home advantage taking into account the UEFA ranking and described home advantage in the most powerful leagues of Europe.

## Material and Methods

### Participants

The sample of this study consisted of 111,030 male soccer matches played in 52 of the highest league categories in UEFA countries between 2000 and 2010. Each game was analyzed from both the home team and away team point of view, meaning a total of 222,060 records.

Different league formats exist in the UEFA territory. Some leagues combine the round-robin system with eliminatory systems; in other leagues, an initial league is formed which is followed by other smaller leagues of teams that descend or win the tournament. Eliminatory phases of different competitions are not used, only regular leagues and play-offs with league format. Although the intention of this study was to use a sample from each country, there are some exceptions. In Liechtenstein league competitions are not held, meaning that in the 53 countries of the UEFA there are only 52 leagues. In the Azerbaijan league the 2002/03 season was cancelled, so only 9 seasons were studied. Serbia and Montenegro started their independent leagues in 2006/07 (4 leagues). In the end, the sample was made up of all the league tournaments which took place in UEFA territory in the 21st century (520 examples over 10 seasons).

Some countries (13) organise their competitions in such a way that they start and end the league in the same year, whereas most countries (36) start the season in the fall and finish it in the spring of the following year. This means that the first group of countries, those that count a season from January to December, have a total of 11 leagues whilst the second group have only 10 leagues during the same period.

### Measures

The variables registered were the final results of matches played as a home team and as an away team, the points obtained in the league, the country, the season, the type of competition and the actual UEFA ranking.

The data used was obtained from the UEFA web page (http://en.uefa.com), and two independent web pages Soccer way (http://www.soccerway.com) and futbol 24 (http://www.futbol24.com). The data collected from these 3 sources was compared in an independent way to minimize the number of errors.

### Procedures

The European soccer leagues are based on a “round-robin tournament” system, which means that the same number of games is played away as at home. In this type of competition, the home advantage is quantified by taking into account the number of games won at home expressed as a percentage of the total number of points obtained (Pollard, 1986). When an independent analysis of the home advantage is made for each team, the home advantage is established by comparing the performance obtained when playing at home with the performance obtained when playing away. This analysis uses the percentage of games won at home in relation to the total of games won both away and at home, understanding draws as half victories.

### Analysis

The degree of significance of the home advantage is calculated with the variable of points obtained, taking for granted the truth of the hypothesis that playing at home poses no advantage (Pollard, 1985; 1986), meaning that one out of every two games played at home is won (50%). To make the contrast, the sign test with Wilcoxon range was used. The groups used were formed of 10 countries in a function of decreasing values of the UEFA ranking, with the exception of the last group, formed of 12 countries (from 41 to 52), called Group A, B, C, D and E, respectively.

In order to compare home advantage between the countries of one group or between the different groups the Kruskal-Wallis test was used.

The bivariate correlations of Pearson were used to establish the level of association between the numbers of points obtained with the advantage of playing at home. Also the Spearmen bivariate correlations were used to assess the association between the classification of a team in a league and the UEFA ranking with the advantage of playing at home.

## Results

The analysis of the sample used reveals a home advantage of 55.6±0.19 in the highest categories of European soccer, and it is significant in all cases analyzed, except for the season 11. The results are shown in Table 1.

### Home advantage evolution in UEFA territory football

A decrease in the tendency of home advantage can be observed between the years of 2000 and 2010 in the UEFA territory soccer leagues. The magnitude of the decrease exceeds two percentage points.

### Home advantage in different countries in the UEFA territory

A significant home advantage was found in 32 (61.5%) of the 52 UEFA territory countries (p<0.05). The most powerful leagues in Europe have statistical significance in home advantage with values between 55 and 56. After this first group, there is a series of countries where home advantage is more heterogeneous: in some nations the existence of home advantage exists and the variability is higher than in the first group. The home advantage reaches a maximum of 76.10 points (Bosnia-Herzegovina) and a minimum of 50.03 (Republic of Ireland). Some cases of disadvantage of playing at home were found (Lithuania, Latvia, Estonia, Wales, Malta, Northern Ireland, Andorra and San Marino). The minimum value observed in San Marino is 45.52 (Table 2).

### Home Advantage and 2010/11UEFA Country coefficients

The UEFA countries were classified by the 2010/11 UEFA Country coefficients in groups of 10 countries. This way, Group A is formed by the ten countries with the highest coefficients. Group B is formed by countries with rankings between 11 and 20, and so on down to Group E, formed by countries between 41 and 52.

The home advantage of each country in each group was compared in order to assess the intragroup homogeneity, obtaining a large homogeneity in Group A (0.974). The rest of the groups show a significant heterogeneity (p<0.001). Significant differences were also found between the home advantage of the groups (p<0.001) (Table 3).

In all countries of Group A, the home advantage is significant with minimum oscillations in its values. In the last ten years, the home advantage decreases by 1.8 points. Between the ten countries of the group, a variation of 1.26 points in home advantage was found. In Group B, 60% of the countries showed the existence of home advantage with more oscillations.

During the decade of the study, home advantage has decreased by 2.15 points. The variation between countries is 6.96 points. In Group C, the percentage rose to 80% and the variation of home advantage is maximum between countries (24.72 points). Home advantage decreased by 1.98 points during the ten years of the study. In Groups D and E, the percentage fell to 40% and 33%, respectively. The variations of home advantage between these countries were high (14.14 and 17.66, respectively). Between 2000 and 2010, home advantage decreased by 0.08 points in Group D and 1.97 points in Group E (Table 4).

**Figure 2:**
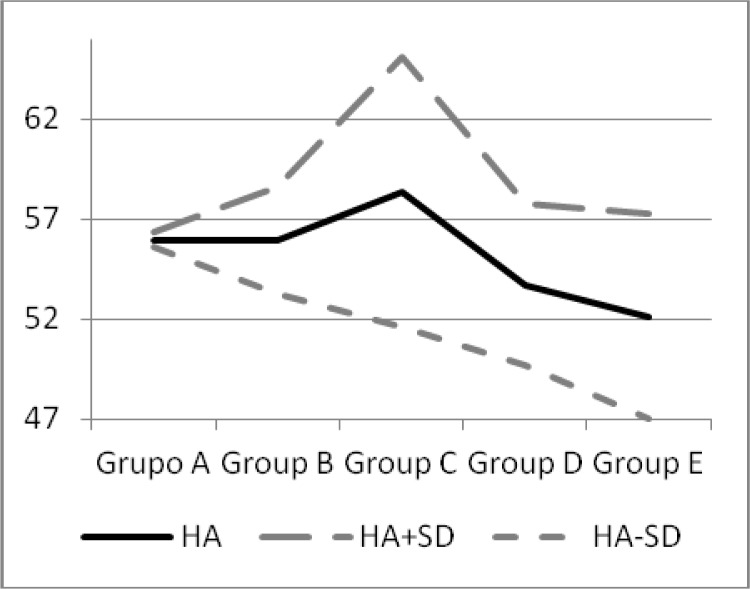
Home advantage according to the group levels established by the UEFA Ranking

The top 20 ranked teams in the UEFA Ranking (Groups A and B) have a very similar home advantage, though the dispersion in Group B is greater than in Group A. After the first 20 leagues, the variability in home advantage increases, reaching 58.35 points in Group C and decreases to 52.12 in Group E (Tables 3 and 4, Figure 2)

An association exists (p<0.001) between home advantage and the UEFA Ranking, therefore it is very weak (0.056).

### Home Advantage, Season Rank and Total points

Both the classification of a team and the number of points won has a significant association (p<0.001) with home advantage. The points obtained by a team are positively associated with home advantage (0.721). The classification of a team in a league has an inverse association with home advantage and a value of −0.674.

## Discussion

The objective of the present study was to analyse the behaviour of home advantage in UEFA-zone soccer in a complete sample: all the leagues in the first decade of the 21st century. According to the results obtained, home advantage of 55.6 has been found in the highest soccer categories of Europe, being significant in every season, except for the season 11, as this last season was made up of only 13 of the 52 countries analyzed, those nations whose seasons coincide with the natural year as opposed to the majority, who play from late summer to the following spring.

Pollard (1986) showed values that oscillated between 53.6% in baseball and 65.5% in North American Soccer, 63.3% for basketball and 63.9% for European soccer. Later on, Pollard (2006b) proved the existence of variations in home advantage in the same sport between different countries, which explains factors such as size of the country, producing longer journeys or factors such as territoriality, defined by Neave and Wolfson (2003).

More recent studies, like the one by Pollard and Pollard (2005) establish for football and basketball values of 60% and for ice hockey 55%, values very similar to the ones found in this study (55.6%). Clarke (2005) found a value of 59.9% in Australian soccer, while García et al. (2009) found home advantage of 55.2% in Spanish basketball and finally, Gutiérrez et al. (2012) found a value of 61.3% in Spanish handball.

The evolution of home advantage during the first decade of the 21st century decreases by two percentage points. This drop seems to indicate that professional teams are progressively overcoming home advantage, decreasing the disadvantage of playing away. But the tendency of this decrease has been different. In the first five-year period of the century, the decrease of home advantage was 1.88 points, while in the second part of the decade, the drop was only 0.2 points, which seems to indicate that home advantage stabilizes at around the value of 54.8.

The degree of evolution of home advantage agrees with the conclusions of Pollard (1986), because as the seasons advance, home advantage declines. In the Pollard study, home advantage changes from 67.9% in the first half of the period to 63.9% almost 100 years later, while in the present study, the drop is more or less 2 points, but it is also true that the Pollard’s study is of a whole century while this study only considers a decade.

For the analysis of home advantage with regard to the quality of teams, 5 groups of 10 teams were established, except for group E, which had 12 teams. To establish this classification, the Country coefficients of UEFA for the 2010/11 season were used: in leagues of group A, the percentage of nations with a home advantage is 100%. In group B, the percentage is 60%, in group C, the percentage increases considerably, reaching 80% of the countries. In groups D and E, which are formed of the 22 countries with the lowest UEFA ranking, there is a low percentage of countries with a significant home advantage (40% and 33%, respectively). Except for group C, there is a tendency towards a decline in the percentage of nations with a significant home advantage in line with the Country coefficients, which is an indicator of the level of competition.

If we focus on the analysis of the top five, we can see that the first five countries (England, Spain, Germany, Italy and France) have a very similar home advantage, as their scores hardly oscillate more than 1.3 points. In other countries, the rest of the groups prove to have an important increase in their heterogeneity values, oscillating between 76.10 (Bosnia-Herzegovina) and 50.03 (Republic of Ireland), even reaching negative values in a few countries, which means that for them there is a disadvantage of playing at home.

When taking into account the influence of the level of the team, the home advantage shows a significant association as there is a positive relation between the points won by a team and home advantage (0.721). The classification of a team in its league has an inverse association with home advantage (−0.674). These results contradict the study of Morton (2006) in rugby and Jacklin (2005) as both concluded that there were no differences in home advantage and the level of the participating teams. Differences also exist between the results of this study and those of Bray (1999) in ice hockey, as he finds that home advantage is similar for all teams independent of the quality of the team. It is necessary to highlight the fact that in ice hockey, the possibility of obtaining a draw is lower than in football. In the matches analyzed by Bray over 20 years, only 13% finished in a draw, while in the present study the percentage is 23.9% of the games analyzed.

However, other studies have obtained results similar to those of this research. The analysis of the category variable coincides with the conclusions of Pollard (1986), as in both studies, the lower the team’s category, the higher the home advantage. This finding could be explained by the fact that teams in lower categories suffer difficulties such as uncomfortable journeys, players having to work or study, lower level of the players in these leagues, or other factors like local pressures. The same conclusion was obtained by Sánchez et al. (2009), who compared home advantage in the two highest categories of Spanish soccer and concluded that home advantage was higher in the first category competition. Finally, similar associations were found by Gutiérrez et al. (2012) in Spanish handball.

## Conclusions

Fifty-two of the fifty-three countries that make up the UEFA territory have league competitions. Only in 32 of them there was a significant home advantage in league competitions at the highest level. In the first decade of the 21st century it was 55.59±0.20.

The countries that made up group A through their UEFA Ranking have a greater homogeneity when taking into account home advantage. In the rest of the groups, home advantage is much more heterogeneous.

The teams with a better classification and more points have better home advantage values.

## Figures and Tables

**Figure 1:**
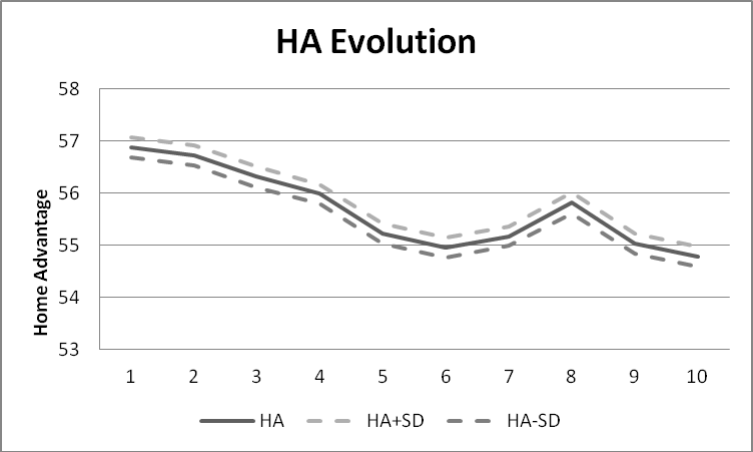
Home advantage evolution in the highest categories of UEFA football

**Table 1:** Home advantage (HA) in the highest categories of UEFA football

	Home Record				HA			

Years	Leagues	Played	Won	Drawn	Lost	%	SD	Signification^1^

2000–2010	520	111030	52853	26609	31568	55.59	0.20	<0.001

Season	Leagues	Played	Won	Drawn	Lost	%	SD	Signification^1^

1	50	10466	5145	2424	2897	56.9	0.20	<0.001
2	50	10523	5142	2481	2900	56.7	0.20	<0.001
3	49	10223	4970	2361	2892	56.3	0.20	<0.001
4	50	10475	5036	2485	2954	56	0.19	<0.001
5	50	10855	5120	2626	3109	55.2	0.19	<0.001
6	50	10920	5136	2595	3189	55	0.20	<0.001
7	52	10929	5096	2802	3031	55.2	0.19	<0.001
8	52	11390	5443	2742	3205	55.8	0.20	<0.001
9	52	11450	5375	2779	3296	55	0.19	<0.001
10	52	11498	5378	2762	3358	54.8	0.20	<0.001
11^*^	13	2301	1012	552	737	52^*^	0.19	<0.146

1 Significance of the Wilcoxon range sign test.

* Season 11 is completed by only 13 of 52 countries.

The HA is not representative of the territory countries of UEFA, because of the 13 countries, only one of them is in the top ten (Russia in 7th position) and the rest of the countries are ranked between 23 and 50.

**Table 2:** Home Record, home advantage and Ranking in the highest categories of UEFA football

	Home Record				Home Advantage						
Country	Leagues	Played	Won	Drawn	Lost	1st Season	Last Season	%	SD	Sig.^1^	Ranking^2^
England	10	3800	1792	978	1030	57.28	59.21	55.74	0.16	**<0.001**	85.785
Spain	10	3800	1761	1049	990	61.05	59.39	55.54	0.14	**<0.001**	82.043
Germany	10	3060	1453	764	843	59.80	50.22	55.81	0.15	**<0.001**	69.436
Italy	10	3504	1626	1013	865	56.32	57.89	56.04	0.15	**<0.001**	60.552
France	10	3652	1719	1066	867	58.17	55.61	56.80	0.13	**<0.001**	53.678
Portugal	10	2796	1315	744	737	58.93	52.78	55.90	0.16	**<0.001**	51.196
Russia	11	2640	1224	741	675	56.25	55.42^*^	55.72	0.15	**<0.001**	44.707
Ukraine	10	2284	1091	572	621	55.31	56.67	56.12	0.18	**<0.001**	43.883
Netherlands	10	2754	1342	619	793	55.23	56.75	56.22	0.18	**<0.001**	40.129
Turkey	10	3059	1453	745	861	57.49	53.49	55.62	0.16	**<0.001**	35.050
Greece	10	2378	1182	592	604	61.94	58.99	58.00	0.20	**<0.001**	34.166
Denmark	10	1980	871	503	606	49.66	50.17	52.46	0.18	<0.136	30.550
Belgium	10	2984	1432	726	826	57.52	54.80	56.10	0.17	**<0.001**	27.000
Romania	10	2664	1365	652	647	64.86	54.14	59.40	0.16	**<0.001**	25.824
Scotland	10	2491	1102	583	806	51.32	50.44	52.44	0.26	<0.776	25.141
Switzerland	10	1960	965	474	521	60.11	60.37	57.30	0.17	**<0.001**	24.900
Israel	10	2058	898	544	616	48.15	51.21	52.45	0.19	<0.140	22.000
Czech Republic	10	2400	1183	681	536	62.08	54.58	58.75	0.14	**<0.001**	20.850
Austria	10	1800	895	457	448	59.63	59.26	58.19	0.15	**<0.001**	20.700
Cyprus	10	1927	896	426	605	54.03	53.82	53.87	0.23	<0.056	18.124
Bulgary	10	2196	1196	430	570	63.74	54.58	60.99	0.20	**<0.001**	17.875
Croatia	10	2034	1091	467	476	57.81	60.00	61.29	0.18	**<0.001**	16.124
Belarus	11	2249	1012	511	726	54.17	51.01^*^	52.57	0.19	<0.099	16.083
Poland	10	2268	1096	576	596	58.06	54.03	56.79	0.17	**<0.001**	15.916
Slovakia	10	1918	984	476	458	60.00	59.43	59.58	0.16	**<0.001**	14.499
Norway	11	2118	1012	529	577	53.30	59.03^*^	56.11	0.15	**<0.001**	14.375
Serbia	4	768	360	203	205	51.77^**^	58.75	55.69	0.16	**<0.027**	14.250
Sweden	11	2176	960	579	637	48.90	57.08^*^	52.99	0.16	**<0.025**	14.125
Bosnia-Herzegovina	10	2762	1953	447	362	70.27	71.94	76.10	0.15	**<0.001**	9.124
Finland	11	1958	840	498	620	54.04	45.97^*^	51.38	0.16	<0.301	8.966
Republic of Ireland	11	2010	822	551	637	50.00	48.52^*^	50.03	0.16	<0.914	8.708
Hungary	10	2184	1040	533	611	58.08	55.97	55.75	0.18	**<0.001**	8.500
Moldova	10	1352	601	335	416	51.49	56.39	52.71	0.22	<0.302	7.749
Lithuania	11	1627	673	377	577	46.30	46.42^*^	49.09	0.23	<0.789	7.708
Latvia	11	1431	627	259	545	42.56	47.16^*^	49.85	0.25	<0.898	7.415
Georgia	10	1657	822	337	498	54.04	54.44	56.39	0.21	**<0.002**	6.957
Azerbaijan	9	1624	741	352	531	56.06	49.65	52.85	0.23	<0.227	6.165
Slovenia	10	1856	877	464	515	63.80	48.15	55.59	0.16	**<0.001**	6.124
FYROM	10	1794	1019	346	429	61.17	57.48	63.23	0.20	**<0.001**	5.207
Iceland	11	1116	489	258	369	54.07	48.74^*^	51.52	0.16	<0.350	4.957
Kazakhstan	11	2616	1397	543	676	61.27	54.51^*^	60.32	0.22	**<0.001**	4.374
Montenegro	4	792	398	193	201	61.11^**^	56.90	58.38	0.17	**<0.004**	3.875
Albania	10	1862	1039	412	411	72.16	64.10	63.18	0.16	**<0.001**	3.874
Estonia	11	1640	724	259	657	45.83	48.52^*^	49.41	0.25	<0.827	3.791
Wales	10	2991	1280	646	1065	51.80	49.13	49.99	0.19	<0.842	2.790
Armenia	11	1232	564	219	449	55.65	50.60^*^	51.70	0.25	<0.469	2.583
Malta	10	1320	547	261	512	51.01	50.25	48.03	0.24	<0.523	2.416
Northern Ireland	10	2244	924	513	807	49.07	49.27	48.80	0.19	<0.305	2.249
Faroe Islands	11	1260	572	269	419	53.33	54.07^*^	52.51	0.20	<0.230	1.416
Luxembourg	10	1628	706	346	576	51.77	49.63	50.45	0.20	<0.887	1.374
Andorra	10	816	344	122	350	48.33	42.92	47.14	0.28	<0.310	1.000
San Marino	10	1540	578	369	593	45.02	47.62	45.52	0.21	**<0.036**	0.916

* Last season is #11.

** First season is 2006/07 (#7).

1 Significance of the Wilcoxon range sign test.

2 Country coefficients 2010/11.

The association or country rankings take into account the results of all clubs from each association and are used to determine the number of entries an association is granted for forthcoming seasons (www.uefa.com).

**Table 3:** Home Record and home advantage according to the level of the group by the UEFA Ranking

	Home Record				Home Advantage				Comparison		

Level	Leagues	Played	Won	Drawn	Lost	%	SD	Sig.^1^	Ranking^2^	Intra-group^3^	Inter-group^3^
Group A	101	31349	14776	8291	8282	55.95	0.16	<0.001	1–10	0.974	0.000
Group B	100	22642	10789	5638	6215	55.90	0.20	<0.001	11–20	0.000	
Group C	98	20447	10504	4716	5227	58.35	0.18	<0.001	21–30	0.000	
Group D	103	16651	7711	3812	5128	53.70	0.20	<0.001	31–40	0.000	
Group E	118	19941	9073	4152	6716	52.12	0.23	<0.001	41–52	0.000	

1 Significance of the Wilcoxon range sign test.

2 Values grouped by Country coefficients 2010/11 in tens, forming five levels of categories of UEFA countries.

3 HA of countries in a group (intra) or between groups (inter) using the Kruskall-Wallis test.

**Table 4:** Home advantage variations according to the level of the group by the UEFA Ranking

Level	% sig.HA^1^	HA 1st Season	HA Last Season	Δ HA	Max. HA	Min. HA	Δ HA	%	SD
Group A	100	57.58	55.78	1.80	56.80	55.54	1.26	55.95	0.35
Group B	60	56.93	54.78	2.15	59.40	52.44	6.96	55.90	2.68
Group C	80	57.81	59.79	−1.98	76.10	51.38	24.72	58.35	6.76
Group D	40	53.76	53.68	0.08	63.23	49.09	14.14	53.70	4.02
Group E	33	53.20	51.23	1.97	63.18	45.52	17.66	52.12	5.11

1 Percentage of countries in a group with a significant home advantage (HA).
